# Assessment of headache characteristics in a general adolescent population: a comparison between retrospective interviews and prospective diary recordings

**DOI:** 10.1186/s10194-016-0602-4

**Published:** 2016-02-24

**Authors:** Anne-Berit Krogh, Bo Larsson, Øyvind Salvesen, Mattias Linde

**Affiliations:** Department of Neuroscience, Faculty of Medicine, Norwegian University of Science and Technology, N-7489 Trondheim, Norway; Faculty of Health and Social Science, Norwegian University of Science and Technology, Trondheim, Norway; Regional Centre for Child and Youth Mental Health and Child Welfare, Central Norway, Norwegian University of Science and Technology, Trondheim, Norway; Unit for Applied Clinical Research, Faculty of Medicine, Norwegian University of Science and Technology, Trondheim, Norway; Norwegian Advisory Unit on Headaches, St. Olav’s University Hospital, Trondheim, Norway

**Keywords:** Headache, Epidemiology, Diary, Interview, Questionnaire, Adolescents

## Abstract

**Background:**

Reliable information on headache characteristics, including frequency and intensity, headache-associated impairment, and use of analgesic medications, may depend on the assessment method used. This study analyzed the correlations between headache characteristics determined in structured interviews and those determined in prospective diary recordings kept by adolescents in the general population.

**Methods:**

In this cross-sectional school-based study, a representative sample of 488 adolescents aged 12–18 years were interviewed about headaches experienced during the previous year. Headache diaries for a three-week period were kept by 393 participants: 244 girls (62 %) and 149 (38 %) boys.

**Results:**

Most adolescents (88 %) who reported headaches during their interview also recorded headaches in their diary. In contrast, 51 % of those who reported being headache-free during the last year recorded headaches in their diary. In the interviews, frequent headaches (at least 50 % of days during the last year) were reported by 2.9 % of participants, while 25.5 % reported this headache frequency in their diary. Overall, the ratings of headache frequency were significantly higher in diaries than in interviews (*p* < 0.001). Significant but low correlations were observed between intensity levels reported retrospectively and prospectively (rho = 0.28; *p* < 0.001) and between average scores of headache-related impairment reported retrospectively and prospectively (rho = 0.35; *p* < 0.001). The use of medications by those who reported one or more current headache disorder during their interview was significantly lower in prospective recordings than in the retrospective interview estimates (*p* < 0.001).

**Conclusions:**

There is inconsistency in the estimates of headache characteristics between retrospective reports and diary recordings. A comprehensive picture of headache complaints among adolescents may be better obtained through a combination of prospective diary recordings and interviews by school health and clinical services.

## Background

Headache disorders are among the most common health problems in adolescents [1]. These include primary headaches, with tension-type headache (TTH) and migraine being the most common, and secondary headache disorders [2, 3]. Headache disorders in school children often lead to reduced quality of life and poorer psychosocial function in schoolwork, leisure activities, and social relations [4, 5], as well as to psychological problems, such as depression and anxiety [6, 7]. Studies monitoring the severity of headache disorders and the response to treatment usually include the frequency of attacks as their main outcome criterion. Outcomes of previous studies suggest that prospective recordings in diaries may provide a more reliable and comprehensive picture of headache occurrence in young people than retrospective assessments based on questionnaires and interviews [8–10].

Most previous clinical studies comparing retrospective and prospective data used sample sizes ranging from 52 to 214 participants [11–15]. Community-based surveys have also yielded somewhat inconsistent results. For example, a Dutch study with children and adolescents aged 9–16 years found that questionnaires overestimated headache intensity and duration when compared with diary recordings, whereas the estimates were equal for headache frequency [8]. A recent Swedish school-based study with adolescents aged 12–18 years reported that questionnaires overestimated headache intensity and underestimated headache frequency and duration when compared with diary recordings [7]. These earlier studies did not include all types of headache. For example, the Dutch study only included school children who reported a headache frequency of at least once a week [8], and the Swedish study excluded adolescents who considered their headaches to be disease-related (approximately one-third of those screened) [7].

The relative paucity of community surveys comparing retrospectively-obtained information and prospective diary recordings of headache characteristics among adolescents suggests further comparisons are needed. Therefore, the present study investigated the agreement between these two sources of information in determining the frequency and intensity of headaches, headache-associated impairment, and the use of medications to treat headache among a sample of adolescents in the general population. In addition, this study analyzed the sensitivity, specificity, and positive and negative predictive values of agreement between retrospectively-reported headaches experienced at least every other day, frequent medication, and prospective information recorded in headache diaries.

## Methods

### Sample selection and recruitment

All secondary and high schools in the county of South-Trøndelag in Norway were surveyed, with 899 eligible students invited to participate using stratified cluster sampling. Randomization was stratified by school location (see below) and age/grade: grade 8 (ages 12–13 years), grade 10 (ages 14–15 years), and grade 12 (ages 16–18 years). The sample distribution corresponded to the distribution of the county’s population: 59 % of students were from urban areas, 28 % were from the inland/mountain region, and 13 % were from coastal municipalities.

At the time of the study, there were 74 secondary and high schools in South-Trøndelag, 13 of which were invited to participate. As there were large differences in school sizes, with more students attending schools in urban than in rural areas, more rural schools were included to obtain the required number of participants. Of the 13 invited schools, four were located in urban areas and nine in rural (inland/mountain or coastal) areas. Seven of the initially invited schools declined to participate and were replaced by seven other schools. The four participating urban schools included two secondary schools (participants in grades 8 and 10) and two high schools (participants in grade 12). The nine participating rural schools included four secondary schools and one high school from the coastal region, and three secondary schools and one high school from the inland/mountain region. Details of the recruitment procedure have been described elsewhere [16, 17].

### Data collection

This study was conducted in two phases. Initially, each adolescent participated in a face-to-face, structured interview with a single interviewer (first author). All interviews were conducted in a suitable room at the participating schools during a regular school day, with interviews lasting 20 minutes on average. A questionnaire was completed during each interview. Immediately afterward, participants were asked to keep a prospective headache diary for a three-week period. Two data collection procedures were used for diaries, one based on paper recordings and the other internet-based [17]. Data were collected from March 2012 to February 2013.

### Questionnaire

The questionnaire used in the interviews was developed for school-based studies of headaches in Sweden [9, 18] and translated into Norwegian. As an opening question, each participant was asked whether he/she had experienced a headache during the previous year. Adolescents who answered “Yes” were asked to report their usual headache frequency as “<1 day/month,” “1–3 days/month,” “1–3 days/week,” or “Every other day or more often.” Participants were also asked if they had experienced more than one type of headache during the previous year. Those who answered “Yes” were asked about the characteristics of and symptoms associated with each headache type. Specifically, participants were asked to report the frequency, intensity (three-point scale: 1 = mild, 2 = moderate, 3 = severe), and duration of episodes, as well as accompanying symptoms.

Participants were asked to complete the Pediatric Migraine Disability Assessment (PedMIDAS). This scale was developed to assess disability in children and adolescents with chronic pain, including headache, and has been used in clinical studies and epidemiological surveys [19–21]. The PedMIDAS consists of six questions addressing the number of days totally or partially lost owing to headache in the last three months across three domains (school, home, and social activities). A total score was calculated for each participant by summing the number of days reported for each item for all headaches [20].

Participants were asked if they had experienced headaches (of any type) for at least 15 days per month during the previous three months. They were also asked about the frequency analgesic medications were used, with response options being “Never,” “1–9 days/month,” and “10 days/month or more often.” Adolescents who used medication were asked the names of those medications. Special consideration was made during the interview to avoid alerting participants to the concept of medication overuse headache (MOH), to avoid influencing their reports of medication use during the next phase of the study.

Headaches were classified as episodic migraine with or without aura, chronic migraine, probable migraine, episodic TTH, infrequent TTH or frequent TTH, chronic TTH, or MOH, according to the International Classification of Headache Disorders (ICHD-3 beta) [22]. Probable TTH was combined with definite TTH. MOH was defined as having any headache (including the most bothersome headache) for more than half of the days during the previous three months and treating it with medication on at least 10 days per month. A headache that did not meet any of the ICHD-3 beta criteria was labeled “unclassifiable.”

The headache diaries included two intensity items: (1) “How intense was your worst headache today?”, and (2) “How intense was your headache on average during the day?” Both items were rated on a 0–10 numerical rating scale (NRS), with 0 = No pain and 10 = Worst imaginable/unbearable pain. Other items included: “How did you function in your daily activities today?” (rated on a 0–3 scale: 0 = No difficulties, 1 = Minor difficulties, 2 = Medium difficulties, and 3 = Major difficulties); and “Did you take any acute medications to treat headache today?” (No/Yes).

A similar paper-based diary (0–5 scale) was originally developed for adults with headaches [23] but has been widely used in intervention studies with adolescents experiencing frequent headaches [24]. Participants who filled out their diary via the internet and who had not logged on before 9 PM each day were sent reminders through a short text message service (SMS). Those who used a paper-based diary were reminded once a week.

### Categorization of headache frequency and medication use

Having a headache was defined as an NRS score of ≥1. For comparisons between questionnaire data, raw scores of headache frequency obtained from the diaries were categorized into three levels: ≤10 %, 11–49 %, and ≥50 % of the days. The corresponding levels and cutoff points in the questionnaire were: <3 days a month (≤10 %), 1–3 days a month or 1–3 days a week (11–49 %), and at least every other day (≥50 %).

The raw scores for medication use in the diary recordings were categorized as “Never,” “Use medications but not very frequently” (at least once but less than 33.3 % of the days in diary recordings), and “Use medications frequently” (33.3 % or more of the recorded days).

### Statistical analysis

Descriptive statistics with means, standard deviations (SD), and percentages with 95 % confidence intervals (CI) were computed. The sensitivity, specificity, positive predictive value (PPV), and negative predictive value (NPV) of interview information for having any headache, headaches ≥50 % of the days, and very frequent medication during the previous year were calculated relative to diary determinations of the same parameters. Kappa statistics with 95 % CI were used to assess the agreement between diary and interview data. Spearman’s rank correlation coefficient (rho) was used to estimate relationships between ordinal variables. Independent t-tests and analysis of variance were used to estimate differences between group means. Differences between groups were analyzed with the McNemar–Bowker test of cell proportion symmetry. Statistical significance was set at a two-tailed *p*-value less than 0.05. All statistical analyses were performed with SPSS Statistics for Windows version 22 (IBM SPSS Statistics for Windows, Version 22.0. Armonk, NY: IBM Corp.).

## Ethics

The research protocol was approved by the Norwegian Ethical Committee for Medical Research. Informed consent was obtained from each participant and documented in accordance with Ethical Committee requirements. Parental consent was obtained for participants younger than 16 years old.

## Results

### Study sample

**Fig. 1:**
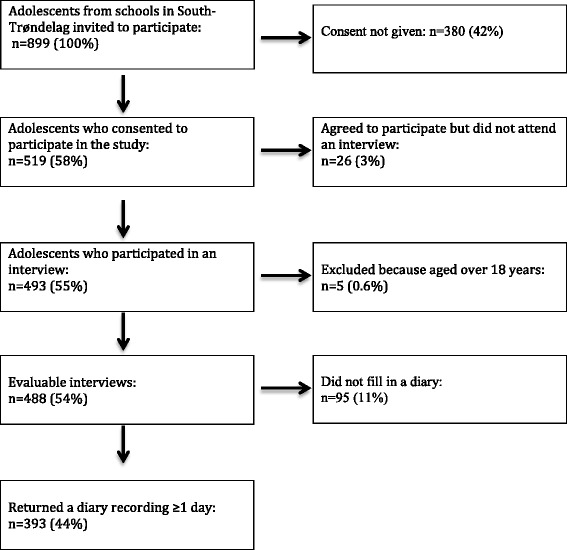
Flow of participants through the study

The flow of participants through the different stages of the study is presented in Fig. 1.

Of the 488 adolescents aged 12–18 years who were interviewed, 276 (57 %) were girls and 212 (43 %) were boys. No participants were disqualified because of lack of internet access. Of the participating adolescents, 393 (81 %) returned diaries: 244 (62 %) girls and 149 (38 %) boys, with an average age of 15.1 ± 1.7 years. Of those who filled out diaries, 214 (54 %) used paper-based and 179 (46 %) used internet-based diaries. Paper-based diaries covered an average of 15.9 ± 8.9 days and internet diaries an average of 12.9 ± 6.9 days over the three-week period.

### Having headaches

Of the 393 participants who completed the interview and diary, 346 (88 %) retrospectively reported headaches in their interviews. Of these, 310 (90 %) reported only one type of headache, and 36 (10 %) reported more than one type. In total, 206 (53 %) were classified as TTH, 130 (33 %) as migraine or probable migraine, one (0.3 %) as MOH, and 11 (3 %) as unclassifiable. Of those who reported more than one type of headache, 31 (86 %) reported TTH as the second headache type and five had migraine or probable migraine.

Of the 346 participants who reported they had experienced headaches during the previous year, 304 (88 %) also documented headache occurrence in their diary. In contrast, of those who did not report a current headache disorder in their interview, 51 % recorded headaches in their diary (Table 1). Comparisons of headache frequency levels reported in interviews with the recorded days of headaches in diaries showed fair agreement, with a Kappa value of 0.33 (95 % CI: 0.20–0.45).

**Table 1:** Sensitivity, specificity, and predictive values of retrospective headache reports (last one year) vs. three-week prospective diary recordings

		Diary			
		Headache	No headache	Total	
Interview	Headache	304	42	346	PPV^a^:304/346 = 88 %
	No headache	23	24	47	NPV^b^:24/47 = 51 %
	Total	327	66	393	
		Sensitivity: 304/327 = 93 %	Specificity: 24/66 = 36 %		

Kappa: 0.33 (95 % CI: 0.20–0.45). McNemar–Bowker test of cell proportion symmetry: *p* = 0.019

^a^Positive predictive value

^b^Negative predictive value

Only participants who filled in the diary for ≥1 day are included in the table

### Headache frequency

Overall, the ratings of headache frequency were significantly higher in the diaries than in the interviews (*p* < 0.001). Although 81 % of participants retrospectively reported one type of active headache disorder during their interview and estimated the frequency as ≤10 % of days, only 27 % recorded such levels in their diary (Table 2). In interviews, the number of participants reporting headaches as occurring ≤10 % of the days was significantly higher than in diaries (*p* < 0.001). In contrast, diary reports of headaches on 11–49 % and ≥50 % of the days were significantly (*p* < 0.001) higher than in interviews. There was only a slight agreement between interview and diary reports of having headaches on at least 50 % of days and one or more headache disorders (Kappa 0.14, 95 % CI: 0.05–0.23; Table 3). Seventy four percent of participants who retrospectively reported having headaches on fewer than 50 % of days per month recorded such headaches in their diary (Table 3).

**Table 2:** Headache frequency reported by adolescents retrospectively (last one year) and during three-week prospective diary recordings

Headache frequency (proportion of days)	Interview (*n* = 310)	Diary (*n* = 310)	Overall	Post hoc test
	n (%)	n (%)	*p*-value	*p*-value^a^
≤10 %	252 (81.3)	84 (27.1)	<0.001	<0.001
11–49 %	49 (15.8)	147 (47.4)		<0.001
≥50 %	9 (2.9)	79 (25.5)		<0.001

Only participants who reported having had one type of headache last year in their interview and filled in the diary for ≥1 day are included in the table

^a^McNemar–Bowker test of cell proportion symmetry

**Table 3:** Sensitivity, specificity, and predictive values of headache for ≥50 % of days: retrospective reports (last one year) vs. three-week prospective diary recordings

		Diary			
		Headache ≥50 % days	Headache <50 % days	Total	
Interview	Headache ≥50 % days	10	1	11	PPV^a^: 10/11 = 91 %
	Headache <50 % days	86	249	335	NPV^b^: 249/335 = 74 %
	Total	96	250	346	
		Sensitivity: 10/96 = 10 %	Specificity: 249/250 = 99 %		

Kappa: 0.14 (95 % CI: 0.05–0.23). McNemar–Bowker test of cell proportion symmetry: *p* < 0.001

^a^Positive predictive value

^b^Negative predictive value

Only participants who reported having had a headache (at least one type) in the last year in their interview and who had filled in a diary for ≥1 day are included in the table

### Headache intensity

During interviews, 30 (13.8 %) participants reported mild intensity, 132 (60.6 %) moderate intensity, and 56 (25.7 %) reported severe intensity. The mean ± SD NRS intensity score recorded in the diaries was 1.9 ± 0.83. For those who reported that they had headaches the previous year in their interview and also recorded at least one headache in their diary, there was a significant but low Spearman correlation between questionnaire intensity levels and mean diary NRS intensity scores (rho = 0.28, *p* < 0.001). Participants who reported headaches during the previous year had significantly higher average (t(199) = −6,39, *p* < 0.001) and worst (t(202) = −8.96, *p* < 0.001) intensity levels in their diary than those who did not.

### Impairment

During the interviews, 207 (95 %) participants had a PedMIDAS score lower than 11 (the limit for a definition of mild disability) and only 11 (5 %) had higher scores. In the diaries, 29 (13.3 %) participants recorded no difficulties during the three-week period, 89 (40.8 %) reported minor difficulties, 71 (32.6 %) reported medium difficulties, and 29 (13.3 %) reported major difficulties. Total mean interview PedMIDAS scores showed significant Spearman correlations with average (rho = 0.36, *p* < 0.001) and maximum (rho = 0.25, *p* < 0.001) impairment scores in the diaries.

### Medication use

Of participants who reported one or more current headache disorder during their interview, the frequency of medication use reported in diaries was significantly lower than that reported in interviews (*p* < 0.001) (Table 4). “No use of medication” was significantly more common in diary recordings (*p* < 0.001), whereas “Medication use but not very frequently” was significantly less common in diaries (*p* < 0.001). There was no agreement between information obtained in interviews and in diary recordings for very frequent medication use (Kappa −0.01, 95 % CI: −0.22–0.00). Of the 346 participants who reported headaches in their interview, only three (0.9 %) also reported very frequent medication use, whereas none of these participants documented very frequent medication use in their diary. In contrast, none of those (2.3 %) who recorded they used medication very frequently in their diary reported very frequent medication in their interview.

**Table 4:** Frequency of medication use reported by adolescents: interviews (last one year) vs. three-week prospective diary recordings

Medication frequency	Interview (*n* = 346)	Diary (*n* = 346)	Overall	Post hoc
	n (%)	n (%)	*p*-value	*p*-value^a^
Never	82 (23.7)	198 (57.2)	<0.001	<0.001
Use but not very frequently	261 (75.4)	140 (40.5)		<0.001
Very frequently	3 (0.9)	8 (2.3)		0.23

Use but not very frequently: taking medication on 1 to <33.3 % of all recording days. Very frequent: taking medication on ≥33.3 % of all recording days

Only participants who reported having had headache (at least one type) during the last year in their interview and filled in the diary for ≥1 day are included in the table

^a^McNemar–Bowker test of cell proportion symmetry

### Medication type

In total, 329 participants used acute headache medication, and 171 (52.0 %) reported using only one class of drug: 154 (90.1 %) used paracetamol alone, 18 (10.5 %) used a nonsteroidal anti-inflammatory drug (NSAID), and two (1.2 %) used triptans. Of the 155 participants who used combinations of two classes of acute drugs, 152 (98.1 %) used paracetamol and a NSAID, and three (1.9 %) were treated with paracetamol and codeine. Three participants used a combination of three classes of acute drugs: two used paracetamol, a NSAID, and codeine, and one used paracetamol, a NSAID, and triptans. Only three participants (0.3 %) were on prescriptive prophylactic drug treatment.

## Discussion

This general population-based study of 393 school-aged adolescents investigated the agreement and relationships between retrospective interviews and prospectively recorded diaries over a three-week period to assess headache characteristics, impairment, and medication use. Overall, the findings showed low correlations and agreement between the two data sources for estimates of headache frequency and intensity, impairment, and medication use. The PPV was high, with most adolescents who reported headache during their interview also documenting a headache in subsequent diary recordings. However, the NPV was over 50 %, suggesting that diary data may identify more individuals with headaches than information obtained during a conventional interview. In the present study, more than half of those who did not report any headaches during their interview recorded a headache during the diary period, although these headaches were of low intensity. In a smaller school-based Swedish study with children and adolescents conducted in 2003, about one-third of those who did not report any headache in a questionnaire recorded headache in their three-week diary; these headaches were primarily of low intensity, a finding consistent with the results of the present study.

While the occurrence of low frequency headaches was higher in the interviews than in diary recordings, the occurrence of high frequency headaches was lower in interviews than in diaries. Similar comparisons have been made in four general population-based studies [7–9, 13]. The results of two of these studies, one Dutch [8] and one Finnish [13], are consistent with our findings [9], with the frequency of headache episodes showing good agreement between interview and prospective diary information.

Interestingly, our findings of a higher prevalence of headaches occurring every day or almost every day in diaries than in interviews resulted in a PPV of 91 %. Many participants who did not report such headache levels in interviews reported them in diaries. An NPV of 74 % indicated a high probability that very high headache frequencies will be captured in diaries, despite individuals stating in interviews that they do not have headaches. Further, a specificity of 99 % indicated there were few false positives. This finding has clear implications for the assessment of frequent and very frequent headaches among adolescents in schools and other health care settings.

Our finding of a low but significant correlation between headache intensity reported in interviews and prospective diary recordings is consistent with the results of a recent Swedish study with school children and adolescents [7]. In that study, headache intensity was higher in questionnaires than in diary recordings. Again, the discrepancy in these findings emphasizes the need for both retrospective interview data and prospective diary information.

Severe disability levels due to headache were rare, as shown in both interview and diary reports, and is consistent with findings for adolescents in the general German population [21]. The impairment levels observed in the present study contrasted strongly with those reported on the PedMIDAS for chronic pain, including headaches, in selected samples of adolescents referred to a tertiary clinic [19, 20]. The PedMIDAS categories were originally developed and validated in a clinical sample of children with migraine [19]. Children with chronic headaches scored much higher than children with non-chronic headaches, and higher than the adolescents in the present sample [8]. Thus, impairment levels in a clinical sample were less sensitive in assessing adolescents with very frequent or severe headaches.

Many adolescents who retrospectively reported use of analgesic medications did not confirm this in their diaries. However, this might have been due to the rather limited three-week recording period. Alternatively, the three-week diary period might have been sufficient to capture very frequent use of analgesic medications. Interestingly, none of the adolescents who reported these levels in interviews also documented them in diaries. The eight adolescents who reported very frequent use of analgesics in their diaries did not report this in their interviews.

This study had several limitations. Data in the diaries were not complete, owing to non-adherence to study protocols. This suggests the need for analyses to be based on the percentage of days completed [17]. This might have biased our estimates regarding the proportions of days with headache, because participants may have been more compliant with their diaries on days with than without a headache. Another limitation was our use of different scales for headache intensity and headache impairment in the interviews and diaries, preventing a determination of levels of agreement. The diary recordings were restricted to three weeks, a time period short enough to be subject to external influences of time factors in the school setting (e.g., variations in the burden of academic work and stress load). The limited recording period might have biased our assessments of retrospective reports of headaches experienced during the previous year compared with a somewhat different but adjacent recording period of three weeks. However, the limited recording period chosen for this study was intended to optimize user adherence among adolescents attending regular schools. Such a time period is also thought to be optimal for recordings of frequent headaches [7, 25–27].

A strength of the present study was its inclusion of a relatively large sample representative of adolescents in the general population, enabling us to extrapolate our results to the general community. Another strength was the lack of delay between completion of the interviews and initiation of diary recordings. The study was conducted over a one-year period (two semesters) and was not restricted to a specific school time or activity. This means that recall bias for the last year potentially caused by particularly restful or stressful periods, such as recently having had a vacation or school examinations, were evenly balanced. Our study also included headaches occurring every day or almost every day, and of the chronic tension type, MOH, or chronic migraine [22]. Our definition of medication use on at least one-third of the days was specifically investigated, because of its clinical use as a definition of MOH.

## Conclusions

There was low agreement and correlation of headache characteristics, impairment, and medication use between retrospective questionnaires and prospective diary information. This suggests that a diary given to an adolescent with headaches may provide information complementary to that obtained during a clinical interview. These findings suggest the usefulness of keeping diaries for children and adolescents with recurrent headaches in school health care systems and clinical services.

The findings also suggest a need for additional general population-based studies, addressing agreements between retrospective and prospective reports when assessing various characteristics of head pain, disability, and medication use. Further work is needed to determine the optimal recording timeframe for diary recordings, as adherence rates may decrease over time, thus reducing the validity of estimates. Various types of reminders may also enhance participation rates in similar community-based surveys.

## Abbreviations

ICHD-3 beta: the third International Classification of Headache Disorders (2013), a beta version
MOH: Medication overuse headache
NPV: Negative predictive value
NSAID: Non-steroidal anti-inflammatory drug
PedMIDAS: Pediatric Migraine Disability Assessment
PPV: Positive predictive value
TTH: Tension-type headache

## References

[1] Berg Kelly K, Ehrvér M, Erneholm T, Gundevall C, Wennerberg L (1991). Self-reported health status and use of medical care by 3,500 adolescents in western Sweden. Acta Paediatr Scand.

[2] Laurell K, Larsson B, Eeg-Olofsson O (2004). Prevalence of headache in Swedish schoolchildren, with a focus on tension-type headache. Cephalalgia.

[3] Anttila P, Metsähonkala L, Sillanpää M (2006). Long-term trends in the incidence of headache in Finnish schoolchildren. Pediatrics.

[4] Karwautz A, Wöber C, Lang T, Bïck A, Wagner-Ennsgraber C, Vesely C, Kienbacher C, Wöber-Bingöl C (1999). Psychosocial factors in children and adolescents with migraine and tension-type headache: a controlled study and review of the litterature. Cephalalgia.

[5] Kernick D, Reinhold D, Jl C (2009). Impact of headache on young people in a school population. Br J Gen Pract.

[6] Larsson B (2009) Migrän och spänningshuvudvärk hos barn och tonåringar. (Migraine and tension-type headache among children and adolescents). Studentlitteratur, Lund

[7] Larsson B, Fichtel A (2014). Headache prevalence and characteristics among adolescents in the general population: a comparison between retrospect questionnaire and prospective paper diary data. J Headache Pain.

[8] van den Brink M, Bandell-Hoekstra EN, Abu-Saad HH (2001). The occurrence of recall bias in pediatric headache: a comparison of questionnaire and diary data. Headache.

[9] Laurell K, Larsson B, Eeg-Olofsson O (2003). Headache in schoolchildren: agreement between different sources of information. Cephalalgia.

[10] Lundqvist C, Clench-Aas J, Hofoss D, Bartonova A (2006). Self-reported headache in schoolchildren: parents underestimate their children’s headaches. Acta Paediatr.

[11] Richardson G, McGrath P, Cunningham S, Humphreys P (1983). Validity of the headache diary for children. Headache.

[12] Labbé EE, Williamsson DA, Southard DR (1985). Reliability and validity of children’s reports of migraine headache symptoms. J Psychopathol Behav Assess.

[13] Metsähonkala L, Sillanpää M, Tuominen J (1997). Headache diary in the diagnosis of childhood migraine. Headache.

[14] Lewandowski AS, Palermo TM, Kirchner LH, Drotar D (2009). Comparing diary and retrospective reports of pain and activity restriction in children and adolescents with chronic pain conditions. Clin J Pain.

[15] Heyer GL, Perkins SQ, Rose SC, Aylward SC, Lee JM (2014). Comparing patient and parent recall of 90-day and 30-day migraine disability using elements of the PedMIDAS and an Internet headache diary. Cephalalgia.

[16] Krogh A-B, Larsson B, Linde M (2015). Prevalence and disability of headache among Norwegian adolescents: a cross-sectional school-based study. Cephalalgia.

[17] Krogh A-B, Larsson B, Salvesen Ø, Linde M (2015) A comparision between prospective Internet-based and paper diary recordings of headache among adolescents in the general population. Cephalalgia Online First Service June:1–11 10.1177/0333102415591506 26092285

[18] Laurell K, Larsson B, Eeg-Olofsson O (2005). Headache in schoolchildren: Association with other pain, family history and psychosocial factors. Pain.

[19] Hershey AD, Powers SW, Vockell AL, LeCates SL, Segers A, Kabbouche MA (2004). Development of a patient-based grading scale for PedMIDAS. Cephalalgia.

[20] Hershey AD, Powers SW, Vockell AL, LeCates S, Kabbouche MA, Maynard MK (2001). PedMIDAS: development of a questionnaire to assess disability of migraines in children. Neurology.

[21] Kroner-Herwig B, Heinrich M, Vath N (2010). The assessment of disability in children and adolescents with headache: adopting PedMIDAS in an epidemiological study. Eur J Pain.

[22] Headache Classification Committee of the International Headache Society (2013). The International Classification of Headache Disorders, 3rd edition (beta version). Cephalalgia.

[23] Blanchard EB, Andrasik F (1985). The headache diary. Management of headaches: a psychological approach. Chapter 5.

[24] Larsson B, Carlsson J, Fichtel Å, Melin I (2005). Relaxation treatment of recurrent headache among adolescents: results from a school-based replication series. Headache.

[25] Osterhaus SO, Passchier J (1992). The optimal length of headache recording in juvenile migraine patients. Cephalalgia.

[26] Larsson B, Fichtel A (2012). Headache prevalence and characteristics among school children as assessed by prospective paper diary recordings. J Headache Pain.

[27] Andrasik F, Lipchik GL, McCrory DC, Wittrock DA (2005). Outcome measurement in behavioral headache research: headache parameters and psychosocial outcomes. Headache.

